# Oleate but not stearate induces the regulatory phenotype of myeloid suppressor cells

**DOI:** 10.1038/s41598-017-07685-9

**Published:** 2017-08-08

**Authors:** Hao Wu, Carl Weidinger, Franziska Schmidt, Jacqueline Keye, Marie Friedrich, Cansu Yerinde, Gerald Willimsky, Zhihai Qin, Britta Siegmund, Rainer Glauben

**Affiliations:** 1 Charité - Universitätsmedizin Berlin, Medical Department for Gastroenterology, Infectious Diseases and Rheumatology, 12200 Berlin, Germany; 2 The First Affiliated Hospital of Zhengzhou University, No. 1 Jianshe East Road, Zhengzhou, 450052 Henan Province China; 3 Freie Universität Berlin, Department of Biology, Chemistry, and Pharmacy, 14195 Berlin, Germany; 4 Key Laboratory of Protein and Peptide Pharmaceuticals, Institute of Biophysics, Chinese Academy of Sciences, Beijing, 100101 China; 5 Charité - Universitätsmedizin Berlin, Institute of Immunology, 13125 Berlin, Germany; 6 German Cancer Research Center, 69120 Heidelberg, Germany

## Abstract

Tumor infiltrating myeloid cells play contradictory roles in the tumor development. Dendritic cells and classical activated macrophages support anti-tumor immune activity via antigen presentation and induction of pro-inflammatory immune responses. Myeloid suppressor cells (MSCs), for instance myeloid derived suppressor cells (MDSCs) or tumor associated macrophages play a critical role in tumor growth. Here, treatment with sodium oleate, an unsaturated fatty acid, induced a regulatory phenotype in the myeloid suppressor cell line MSC-2 and resulted in an increased suppression of activated T cells, paralleled by increased intracellular lipid droplets formation. Furthermore, sodium oleate potentiated nitric oxide (NO) production in MSC-2, thereby increasing their suppressive capacity. In primary polarized bone marrow cells, sodium oleate (C18:1) and linoleate (C18:2), but not stearate (C18:0) were identified as potent FFA to induce a regulatory phenotype. This effect was abrogated in MSC-2 as well as primary cells by specific inhibition of droplets formation while the inhibition of de novo FFA synthesis proved ineffective, suggesting a critical role for exogenous FFA in the functional induction of MSCs. Taken together our data introduce a new unsaturated fatty acid-dependent pathway shaping the functional phenotype of MSCs, facilitating the tumor escape from the immune system.

## Introduction

Obesity has been identified as an independent risk factor for a variety of cancers including colorectal cancer^1–3^. However the mechanisms driving this pro-tumorigenic state have not been entirely elucidated. The visceral fat tissue is the source of a number of soluble mediators including cytokines, adipokines as well as chemokines that determine the local milieu. For example the pro-inflammatory milieu in the visceral fat tissue in obesity has been identified as key factor for insulin resistance^4, 5^. Besides the described soluble mediators, the visceral fat tissue is the primary source for free-fatty acids (FFA)^6, 7^. Remarkably, while adipose tissue is the main site of fatty acid synthesis in mammals, tumor tissue itself has been revealed to be a source of FFA suggesting that FFA themselves might have the potential to determine the local milieu and hence tumor growth^7, 8^.

During the last decade, two emerging hallmarks have been added to the classical hallmarks of cancer, namely reprogramming of energy metabolism and evading immune destruction^9^. Here, especially the lipid metabolism of tumor cells has been addressed in several studies and could be identified as crucial factor for further tumor progression^10, 11^. For example, FFA released by human breast cancer tissue sufficed to suppress cytotoxic T cell responses suggesting that FFA can directly modulate the anti-tumor response^8^. Additional data from 1970s indicate that not FFA in general but rather defined FFA are responsible for this observed immunosuppressive effect. Here, an increased number of experimental tumors were observed after an exposure to oleate-enriched diet^12^. Furthermore, an epidemiological study showed that patients within the highest quartile of oleic acid content (>38% of total adipose tissue fatty acids) bear 7.5 time higher probability of metastatic lymph nodes than the patients in the lower quartile (<35% of total adipose tissue fatty acids)^6^.

Which cells represent the primary target for the FFA-mediated effects? A recent study provides evidence that dendritic cells from tumor bearing mice or cancer patients are characterized by high amounts of triglycerides, caused by an increased uptake of extracellular lipids. These dendritic cells were not only characterized by lipid droplets, the accumulation of intracellular FFA, but furthermore lost their ability of cross presentation that ultimately led to tumor progression^13, 14^. These data indicate that myeloid cells represent a target population for FFA.

The heterogeneity of tumor infiltrating myeloid cells link to their contradictory immune function in the tumor microenvironment^15^. Myeloid derived suppressor cells (MDSCs) and tumor associated macrophages (TAMs) represent the two major inhibitory myeloid populations in the tumor. These two subsets share several common mechanisms to regulate T cell responses including NO release, arginine deprivation via arginase, the aggressive activation of indoleamine-pyrrole 2,3-dioxygenase (IDO) and the synthesis of peroxyntitrite (PNT). Besides, a subset of MDSCs (M-MDSCs) differentiate rapidly into TAMs after migrating into tumor site indicating a close correlation between these two cell types^16^. However, which factor(s) derived from tumor milieu leading to the potent suppressive capacity of myeloid cells is still unclear.

Thus in the present study, the MSC-2 cell line as well as primary bone marrow-derived myeloid cells served to elucidate the effect of specific FFA on MSCs function. Our data indicate that in particular sodium oleate, an unsaturated FFA, induces an inhibitory function in both cell line and primary cells. This inhibitory effect was controlled by the amount of intracellular FFA and by droplets formation. Sodium oleate-dependent induction of NO was revealed as the central mechanism mediating this inhibitory function. Thus we here suggest a novel sodium oleate-dependent pathway to induce MSCs.

## Results

### Sodium oleate is sufficient to induce a regulatory phenotype in MSC-2 cells

**Figure 1:**
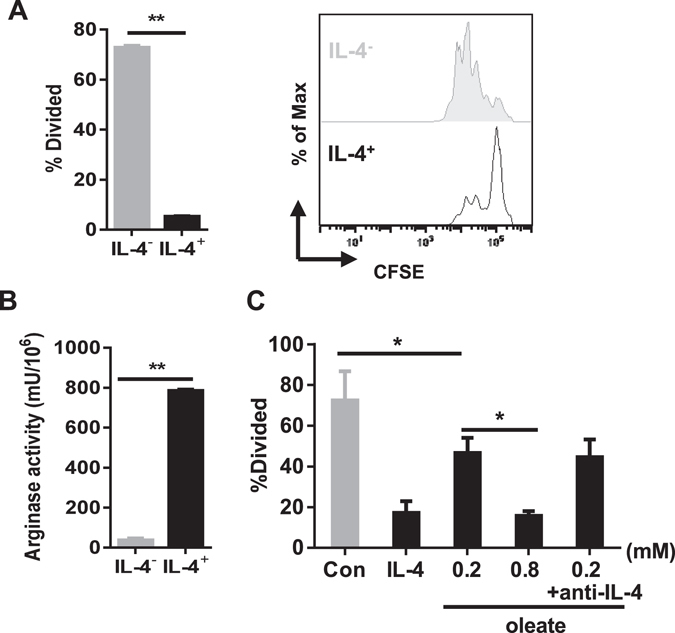
Sodium oleate is sufficient to induce a regulatory phenotype in MSC-2 cells. MSC-2 cells were incubated in the presence or absence of IL-4 (100 ng/ml) for four days as indicated, followed by the analysis of their inhibitory potency: Purified CD4^+^ T cells were co-cultured with MSC-2 cells in the presence or absence of IL-4 in a ratio of 1:5. T cell proliferation was analyzed by CFSE staining after 72 h (**A**). After a four-day culture of MSC-2 cells in the presence or absence of IL-4 (100 ng/ml), arginase activity was determined (**B**). Purified CD4^+^ T cells were co-cultured with MSC-2 cells in the presence or absence of IL-4, sodium oleate (0.2 mM) or a neutralizing IL-4 antibody (10 µg/ml) as indicated. T cell proliferation was analyzed by CFSE staining after 72 h (**C**). Shown is the mean ± SD from two to three independent experiments. *p ≤ 0.05; **p ≤ 0.01.

The MSC-2 cell line served to investigate the regulatory mechanisms of myeloid suppressor cells (MSCs). As described previously^17^, when cultured in the presence of IL-4, MSC-2 cells differentiated into a regulatory phenotype, accompanied by up-regulation of CD80, MHCII, CD4 and CD11b (data not shown). Functionally, these MSC-2 cells inhibited T cell proliferation (Fig. 1A) paralleled by a strong increase in arginase activity (Fig. 1B). Hence in the following experiments IL-4-treated MSC-2 cells served as positive control for regulatory myeloid cell function.

It was previously reported, that accumulation of lipid droplets is paralleled by dendritic cell dysfunction^13^. Free fatty acids (FFA) represent one of the most important sources for the synthesis of intracellular lipid droplets. In the presence of sodium oleate, MSC-2 cells resembled the above-introduced positive control with regard to their suppressive capacity. This regulatory function, as evaluated by suppression of T cell proliferation, could be induced in a dose-dependent manner and was completely independent of endogenous IL-4, since the presence of neutralizing IL-4 antibodies did not abolish the regulatory properties (Fig. 1C). The transition from non-regulatory myeloid cells to functional suppressor cells seemed to be restricted to MSC-2 cells, since the macrophage cell line RAW264.7 did not differentiate into a regulatory phenotype in the presence of sodium oleate (Supp. Figure 1).

### Sodium oleate but not stearate induces a regulatory phenotype in MSC-2 cells

**Figure 2:**
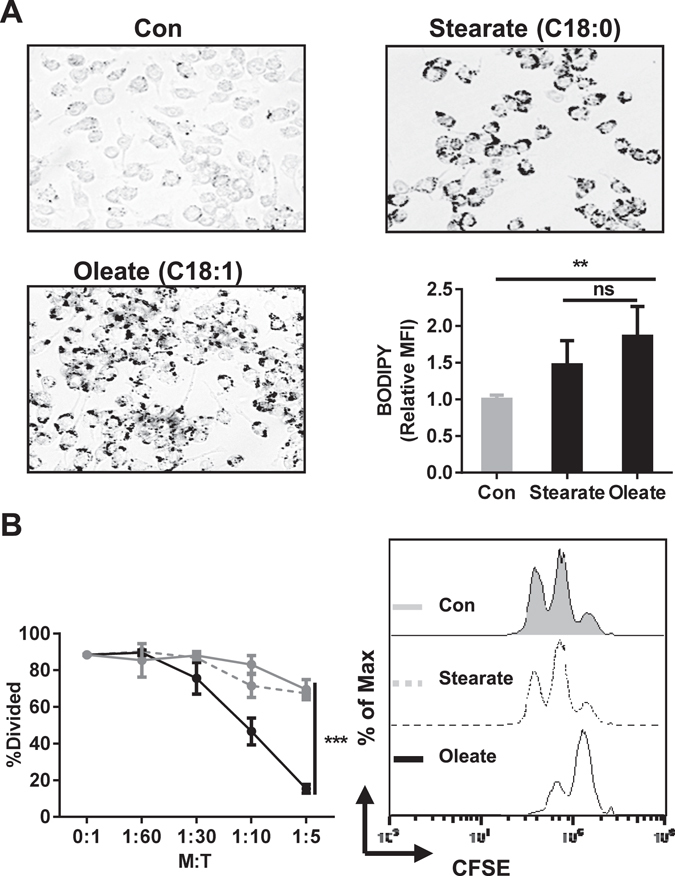
Sodium oleate but not stearate induces a suppressive phenotype in MSC-2 cells. MSC-2 cells were incubated for 24 h with either sodium oleate (0.2 mM) or stearate (0.2 mM). The accumulation of lipid droplets was detected either microscopically via oil-red-O (×20), or by BODIPY staining and flow cytometry (**A**). To assess the inhibitory effect of either sodium oleate or stearate, MSC-2 cells, pre-treated with the respective FFA, were subsequently co-cultured with purified CD4^+^ T cells at the ratios indicated. T cell proliferation was analyzed by CFSE staining after 72 h (**B**). Shown is the mean ± SD from three to four independent experiments. **p ≤ 0.01; ***p ≤ 0.001.

Both saturated and unsaturated fatty acids serve as metabolic components and can be stored as lipid droplets in conditions of nutritional satisfaction^18^. To explore the potential regulatory role of saturated and unsaturated fatty acids, MSC-2 cells were cultured in the presence or absence of either sodium oleate (C18:1) or sodium stearate (C18:0). Even though both fatty acids shared the ability of droplets formation as detected by either oil-red-O or BODIPY staining (Fig. 2A), only the presence of sodium oleate, but not sodium stearate, sufficed to induce the regulatory phenotype as indicated by the inhibitory effect on T cell proliferation (Fig. 2B).

### Sodium oleate-induced regulatory phenotype of MSC-2 cells depends on NO production but not arginase activity

**Figure 3:**
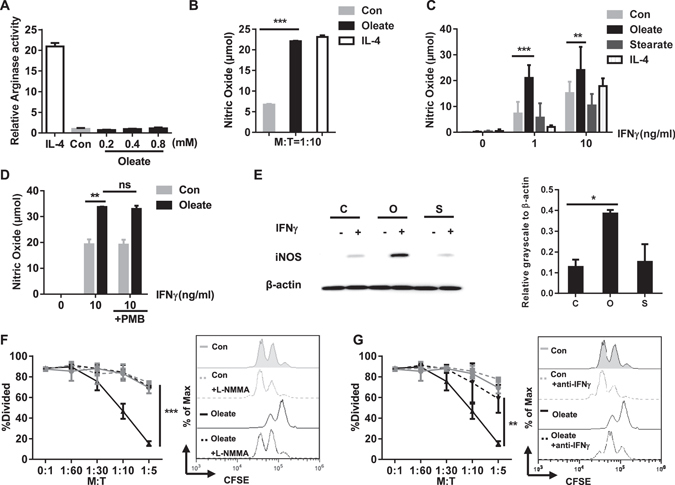
The sodium oleate-induced regulatory phenotype of MSC-2 cells depends on NO production but not arginase activity. MSC 2 cells were cultured in the presence of either IL-4 (100 ng/ml), BSA (Con) or sodium oleate (0.2 mM). After 24 h arginase activity was determined (**A**). MSC-2 cells were co-cultured with purified CD4^+^ T cells for 72 h in the presence of either BSA, sodium oleate (0.2 mM) or IL-4 (100 ng/ml) and NO was measured via Griess reagents in the supernatant (**B**). MSC-2 cells were stimulated with the indicated concentrations of IFNγ for 24 h in the presence of either BSA (Con), sodium oleate (0.2 mM), sodium stearate (0.2 mM), IL-4 (100 ng/ml) and IFNγ as indicated. NO was measured via Griess reagents in the supernatant (**C**). MSC-2 cells were treated with BSA (Con), sodium oleate (0.2 mM) in the presence or absence of IFNγ (10 ng/ml) and polymyxin B (10 µg/ml) as indicated (**D**). MSC-2 cells were pretreated overnight with BSA (**C**), sodium oleate (O) or sodium stearate (S) and subsequently stimulated for 8 h with IFNγ (1 ng/ml). Cell lysates were analyzed by Western blot analysis for iNOS as well as beta-actin expression. Quantitative evaluation in relation to β-actin expression was performed. Shown is the mean ± SD of n = 2 experiments (**E**). MSC-2 cells were co-cultured with CD4^+^ T cells in the ratios indicated and in the presence or absence of BSA (Con), sodium oleate (0.2 mM) and the iNOS inhibitor L-NMMA (2.5 mM) (**F**) or anti-IFNγ (10 µg/ml) (**G**). Cell proliferation was evaluated by CFSE staining after 72 h. Shown is the mean ± SD from two to four independent experiments. *p < 0.05; **p < 0.01; ***p < 0.001.

The suppressive effect of MDSC has been described to rely on two mechanisms: Release of NO to block the activation of the IL-2 signaling pathway in T cells and a boost in arginase activity to exhaust arginine, which is crucial for the synthesis of the CD3ζ chain^19^. To explore the mechanism by which sodium oleate induced the regulatory phenotype, both NO production and arginase activity were analyzed. The presence of sodium oleate did not suffice to elicit arginase activation (Fig. 3A), hence excluding arginase activity as potential mechanism of the regulatory function.

However, in the T cell co-culture system as well as after stimulation with IFNγ, the presence of sodium oleate resulted in a significant increase in NO production (Fig. 3B/C). Unspecific endotoxin effects were excluded by performing the experiment in the presence of the LPS-blocking reagent polymyxin B (Fig. 3D).

In contrast, sodium stearate failed to induce NO production (Fig. 3C). This was further supported by the high expression of iNOS in IFNγ-stimulated MSC-2 cells in the presence of sodium oleate but not sodium stearate (Fig. 3E). In line, the sodium oleate-mediated regulatory effect of MSC-2 cells in the T cell proliferation assay could be neutralized either by L-NMMA, an inhibitor of the NO synthase or in the presence of an IFNγ neutralizing antibody (Fig. 3F/G). In addition, the NO-dependency of this sodium oleate-mediated regulatory function was further supported by comparing MSC-2 and RAW264.7 cells. Here RAW264.7 cells failed to produce NO in the presence of sodium oleate and subsequently failed to suppress T cell proliferation (Supp. Figure 1B/C).

### The suppressive capacity of MSC-2 cells relies on the presence of lipid droplets

**Figure 4:**
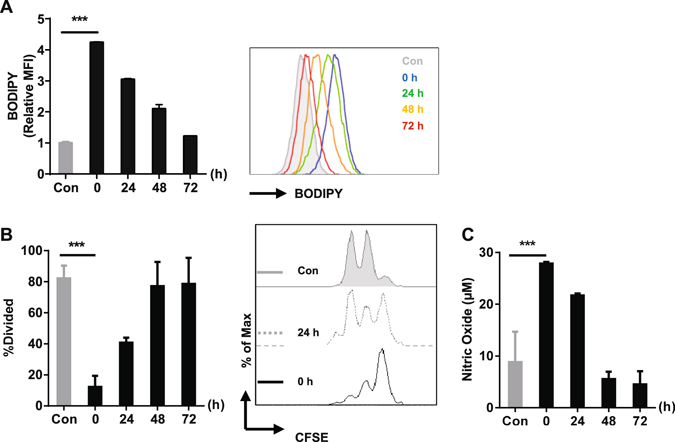
Kinetic of lipid droplets formation and suppressive capacity after sodium oleate treatment. MSC-2 cells were cultured in the presence or absence of sodium oleate (0.2 mM) for 24 h. Cells were washed and left in BSA-medium for up to 72 h and the analysis was performed at the time points indicated. The formation of lipid droplets (**A**) were analyzed by BODIPY staining. The regulatory function of MSC-2 cells from those groups was analyzed via T cell proliferation assay (**B**). Supernatant from the co-culture served for the quantification of NO production (**C**). Shown is the mean ± SD from two to three independent experiments. ***p ≤ 0.001.

Uptake of FFA led to the accumulation of lipid droplets in the cytoplasm. Once sodium oleate had been removed from the medium, MSC-2 cells consumed their lipid droplets within 72 h (Fig. 4A). This loss of lipid droplets, as determined by flow cytometry, correlated with the reduction of NO production (Fig. 4C) and subsequently with the loss of suppressive capacity of these cells in the T cell proliferation assay (Fig. 4B).

### Diacylglyercol acyltransferase-dependent lipid droplets formation controls the effect of sodium oleate on MSC-2 cells

**Figure 5:**
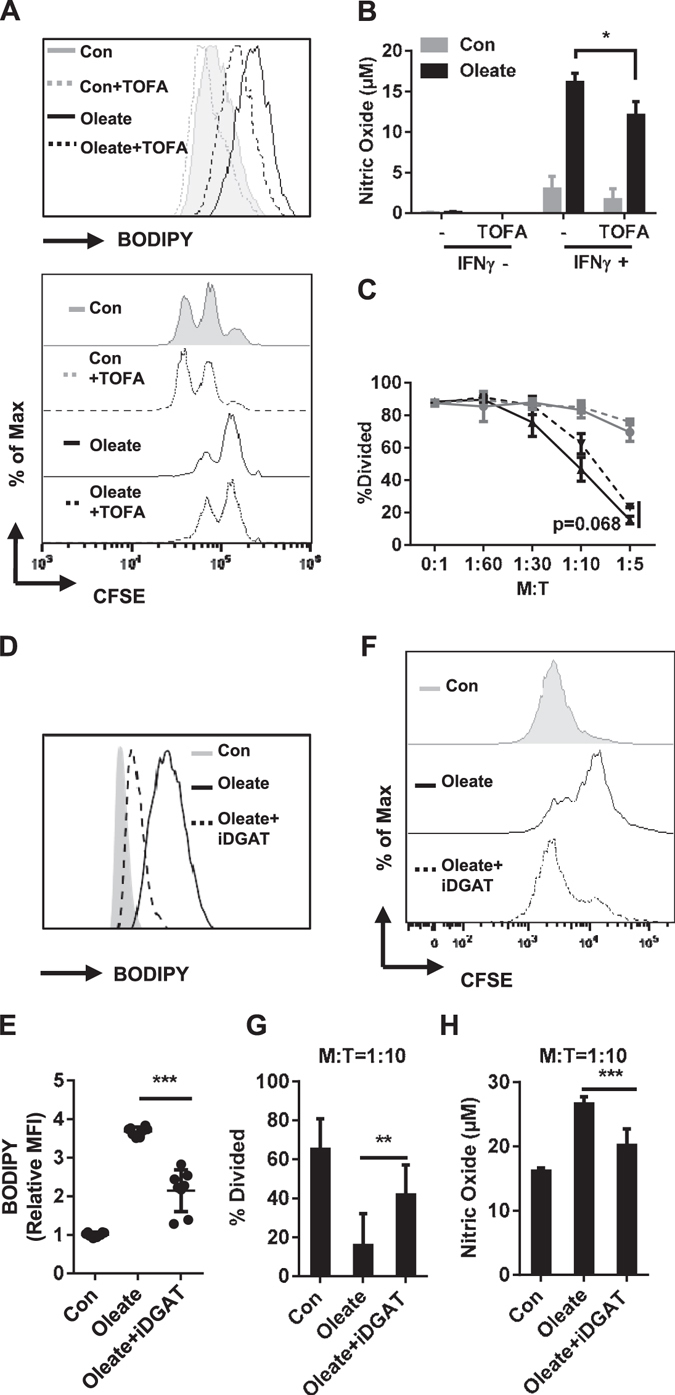
Diglyceride acyltransferase (DGAT) dependent lipid droplets formation controls the effect of sodium oleate on MSC-2 cells. MSC-2 cells were incubated with TOFA (5 µg/ml) in the presence or absence of sodium oleate (0.2 mM). Droplets formation was evaluated by BODIPY staining and flow cytometry (**A**). MSC-2 cells were incubated in the presence or absence of sodium oleate and TOFA as indicated and were subsequently stimulated with IFNγ (1 ng/ml) for 24 h. NO production was determined (**B**). MSC-2 cells were co-cultured with CD4^+^ T cells in the ratios as indicated and in the presence or absence of TOFA (5 µg/ml). Cell proliferation was evaluated by CFSE staining after 72 h (**C**). Combination of DGAT inhibitors was added together with sodium oleate for 24 h, the lipid droplets formation was analyzed via BODIPY staining (**D**, **E**). Functional assay was also performed in the co-culture system with CD4^+^ T cells: the proliferation of T cells (**F**, **G**) and the production of NO in the co-culture wells (**H**) were measured. Shown is the mean ± SD from two to four independent experiments. *p < 0.05; **p < 0.01; ***p < 0.001.

While tumor cells are capable of *de novo* fatty acid synthesis, tumor stroma cells prefer the uptake of exogenous FFA^20^. Acetyl-CoA carboxylase (ACC) catalyzes the irreversible carboxylation of acetyl-CoA to malonyl-CoA, which is the most important element for *de novo* fatty acid synthesis. Here the specific inhibitor of ACC, 5-(tetradecyloxy)-2-furoic acid (TOFA), served to investigate the role of *de novo* fatty acids with regard to the sodium oleate-induced suppressive effect. The presence of TOFA reduced the sodium oleate-induced lipid droplets formation, paralleled by a modest reduction of the inhibitory function of these MSC-2 cells (Fig. 5A/B). These results exclude an exclusive role of *de novo* fatty acid synthesis in mediating the suppressive capacity of sodium oleate-induced MSC-2 cells. The enzymes directly connected to the lipid droplets accumulation are the diacylglycerol acyltransferases (DGAT) 1 and DGAT2. Harris *et al*. proved that the accumulation of lipid droplets in adipocytes was only diminished in DGAT1 and DGAT2 double knockout (KO) but not in single KO mice^21^, indicating a potent compensatory mechanism between the two enzymes. In line, our own data demonstrated that only the combined inhibition of both DGAT by iDGAT (400 nM) sufficed to abolish sodium oleate-induced lipid droplets formation in MSC-2 cells (Fig. 5C/D). In parallel, with lipid droplets formation, the presence of iDGAT impaired the inhibitory function of these cells (Fig. 5E/F) as well as the NO production in the co-culture supernatants (Fig. 5G). These results suggest that not only the presence of distinct free fatty acids in the cytoplasm but also the lipid droplets formation itself mediates the regulatory phenotype of MSC-2 cells.

### Both sodium oleate and sodium linoleate induce a regulatory phenotype in bone marrow-derived myeloid cells

**Figure 6:**
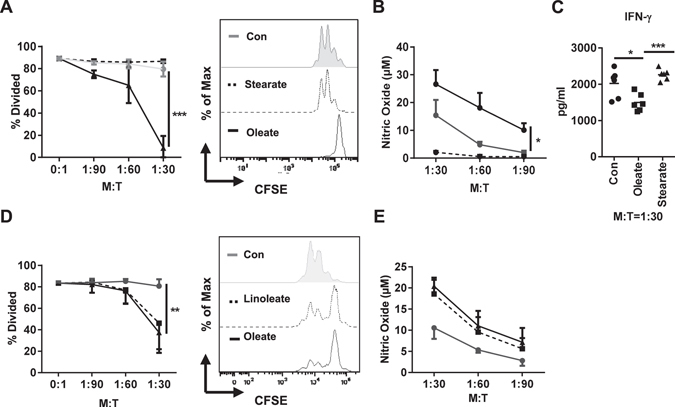
Both sodium oleate and sodium linoleate induces a regulatory phenotype in bone marrow-derived myeloid cells. Bone marrow cells isolated from naïve mice were cultured with GM-CSF (40 ng/ml) and FFA as indicated for 7 days. FFA-treated cells or controls were co-cultured with CD4^+^ T cells in the ratios as indicated. Cell proliferation was evaluated by CFSE staining after 72 h (**A/E**). Supernatants were served for NO analysis (**B/F**) and IFNγ detection (**C**). Shown is the mean ± SD from two to four independent experiments. *p < 0.05; **p ≤ 0.01;***p ≤ 0.001.

As adult primary myeloid cells, suppressive or non-suppressive, were not affected by the presence of sodium oleate (Supp. Figure 2), we chose to differentiate suppressive myeloid cells from bone marrow precursors. Modifying a GM-CSF-based protocol^22, 23^, myeloid cells were derived from the bone marrow of healthy wild type mice as described in the methods section in the presence of either sodium oleate, sodium stearate or BSA as vehicle control. When analyzed in a T cell proliferation assay, the sodium oleate-treated cells confirmed their MSC-phenotype, while the sodium stearate- as well as BSA-treated groups failed to affect T cell proliferation (Fig. 6A). This effect was again paralleled by a significantly enhanced NO production of the sodium oleate-treated cells as compared to the sodium stearate or BSA groups, hence confirming the successful generation of primary MSCs from bone marrow precursors via the presence of sodium oleate (Fig. 6B). Furthermore, the supernatant of myeloid-T cell co-culture system from oleate group contained a dramatically reduction of IFNγ, the dominant cytokine released from activated T cells, validating the regulatory effect of oleate on myeloid cells (Fig. 6C). Here, treatment with the unsaturated fatty acid (18:2) sodium linoleate was equal to the effect induced by sodium oleate: the bone marrow-derived myeloid cells developed an immunosuppressive phenotype (Fig. 6D/E).

### Gr1^−^ but not Gr1^+^ cells exhibit enhanced immunosuppressive capacity after oleate treatment

**Figure 7:**
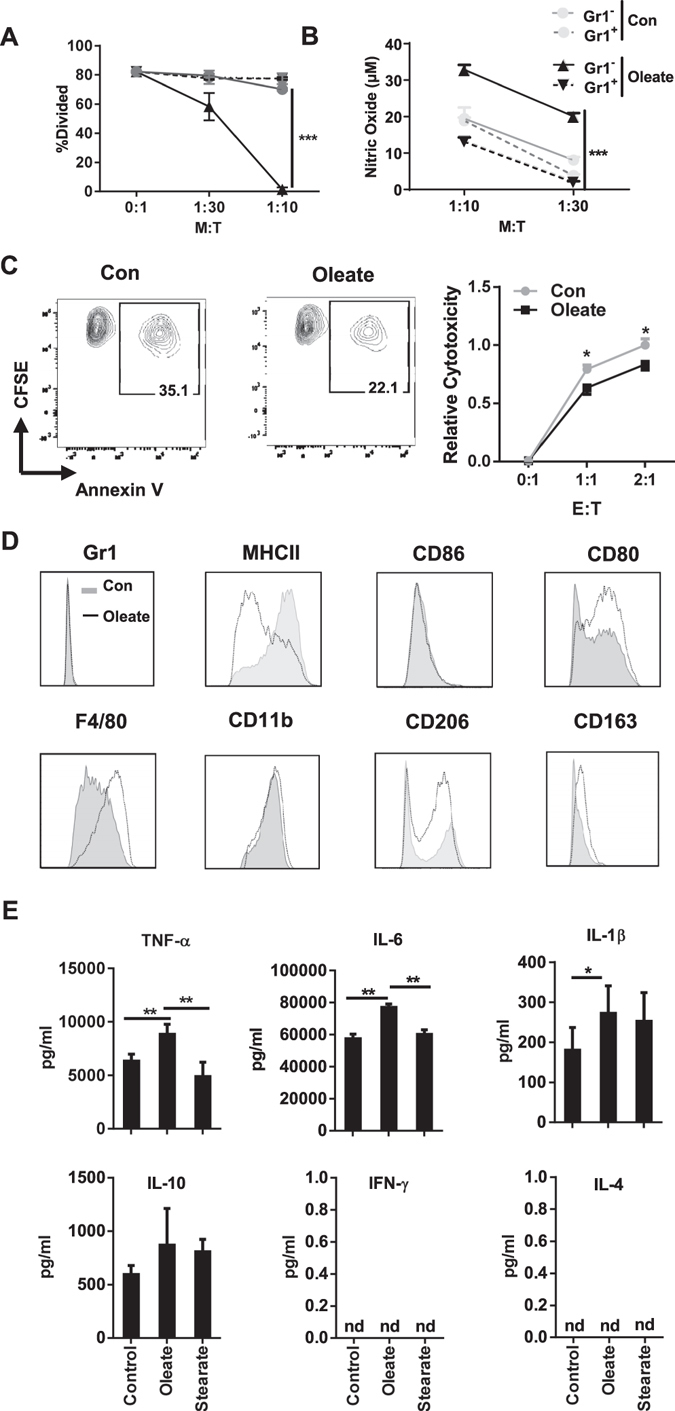
Gr1^−^ but not Gr1^±^ cells exhibit enhanced immunosuppressive capacity after oleate treatment. Bone marrow cells were polarized in the presence of 40 ng/ml GM-CSF and treated with the indicated compounds for 7 days. Gr^+^ and Gr1^−^ populations were purified via beads and co-cultured with purified CD4^+^ T cell in the ratios indicated for functional assay (**A**). NO production from the co-culture supernatant was quantified by Griess reaction (**B**). CTLs were polarized and coincubated with purified Gr1− cells for 18 hours. The suspension cells were harvested and cocultured with CFSE labelled E.G7 OVA cell line for 90 mins. The proportion of Annexin V^+^ cells gated from CFSE^+^ population was used to calculate the cytotoxicity of CTLs (Formula in the Methods section) (**C**). Surface marker panel of Gr1^−^ cells from either control or oleate group was performed via flow cytometry (**D**). Purified Gr1^−^ cells were stimulated by 1 µg/ml LPS in 24 hours and then supernatants were collected for cytokine analysis (**E**). Shown is the mean ± SD from two to four independent experiments. *p < 0.05; **p ≤ 0.01;***p ≤ 0.001.

To identify the suppressive subsets in primary myeloid cells after oleate treatment, Gr1^+^ and Gr1^−^ cells were sorted for functional analysis (Supp. Figure 3). Although Gr1^+^ cells accumulated after oleate treatment (Supp. Figure 4), these cells failed to suppress T cell proliferation in the ratio of 1:10. In contrast, oleate potentiates the inhibitory capacity of Gr1^−^ cells as well as their NO production (Fig. 7A/B). E.G7-OVA cell line was used here to test the impact of polarized myeloid cells on the cytotoxicity of T lymphocytes (CTLs). In contrast to the CTLs coincubated with BSA pretreated myeloid cells, OVA specific cytotoxicity of CTLs was decreased significantly after coculture with oleate pretreated Gr1^−^ populations (Fig. 7C). Phenotypically, Gr1^+^ cells express low levels of CD11b, CD80 and CD86 and are negative for CD11c, F4/80, CD163 and CD206. These results suggested that accumulated Gr1^+^ cells in the oleate group are immature myeloid cells but neither DCs nor macrophages. Notably, the surface marker expression panel of Gr1^−^ cells resembles the one from the MSC-2 cell line, including diminished Gr1 expression and upregulated F4/80. Oleate treatment elevated the expression of CD163 and CD206 paralleled by a reduction of MHCII, indicating a conversion of the myeloid cell phenotype from antigen presenting into immune regulating (Fig. 7D). Cytokine detection via cytometric bead array (CBA) proved an oleate specific induction of IL-6 and TNFα. A significant increasing of IL-1β was induced by both oleate and stearate, although the concentration was negligible compared with either TNFα or IL-6. IL-4 and IFNγ were not detectable in all the groups (Fig. 7E).

### Inhibition of DGAT reverses the induction of a regulatory phenotype on primary cells by sodium oleate

**Figure 8:**
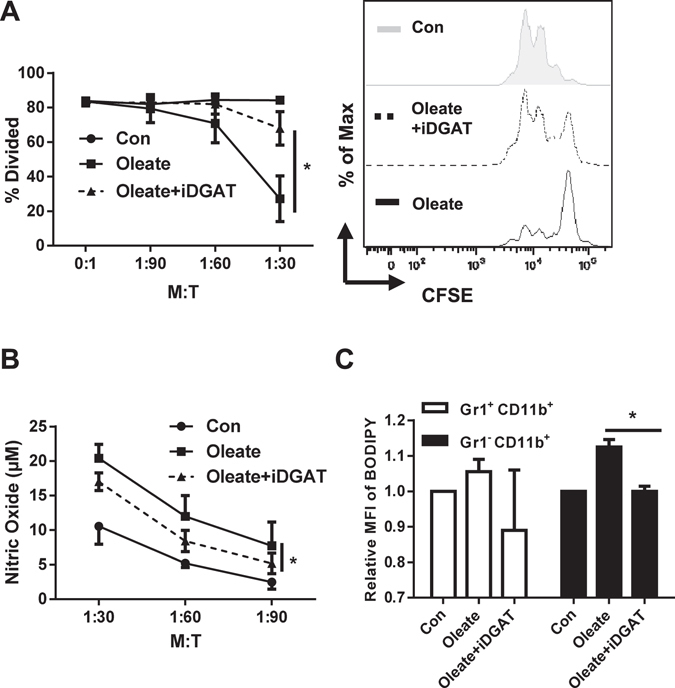
Inhibition of DGAT impedes the impact of sodium oleate on primary cells bone marrow cells. Bone marrow cells were polarized in the presence of 40 ng/ml GM-CSF and treated with the indicated compounds for 7 days. Functional assay was performed via co-culture with purified CD4^+^ T cell in the ratios indicated (**A**). NO production from the co-culture supernatant was quantified by Griess reaction (**B**). BODIPY staining was used to detect the lipid droplets formation in variant subpopulation of primary bone marrow cells (**C**). Shown is the mean ± SD from two to four independent experiments. *p < 0.05.

To validate the effect of DGAT on primary bone marrow cells, iDGAT was added in the presence of sodium oleate to the polarizing cells and the emerging phenotype was analyzed. Again, DGAT function proved to be essential for T cell suppression, NO production as well as lipid droplets formation of the primary MSCs. (Fig. 8A–C). Remarkably, oleate treatment induces lipid droplets formation in Gr1^−^ but not Gr1^+^ cells, which again is impaired by iDGAT treatment effectively. These results validate that oleate represents a functional inducer of the immunosuppressive phenotype, primarily targeting Gr1^−^ cells (Fig. 8C).

## Discussion

Remarkably, different free fatty acids (FFA) fulfill diverse biological functions according to their saturation state. For example, high doses of saturated FFA exhibit a toxic effect on human bone marrow mesenchymal stem cell viability, which can be reversed by adding unsaturated fatty acids^24^. Furthermore the selective inhibition of stearoyl Co-A desaturase, the key enzyme to catalyze stearate into oleate, induces an elimination of human pluripotent stem cells^25^. This phenomenon has been associated with the occurrence of endoplasmatic reticulum stress, which is directly regulated by the ratio of cytoplasmic saturated and unsaturated fatty acids^26^. Focusing on the impact of fatty acids on myeloid cells, it has been described, that palmitate triggers the transformation of macrophages and Kupffer cell into pro-inflammatory cells whereas oleate promotes the expression of anti-inflammatory markers^27^. In addition, oleic and stearic acid induce differential effects also in neutrophils, as indicated by diverse phagocytosis and candidacidal effects^28^. Both fatty acids can be found in tumor tissue in different concentrations. In ovarian cancer tissue, the amounts of stearate and oleate are both about 100 µM, compared to 80 µM stearate and 40 µM oleate in benign tissue^11^. Also, breast cancer tissue shows a 100 fold increase in the release of oleate compared to benign breast tissue^8^. In Ehrlich ascites tumor model, the concentration of FFAs in ascites fluid of tumor bearing mice is about 0.244 mM^7^, containing 32.2% oleate and 12.6% stearate. To induce lipid droplets, a range from 0.2 mM to 0.6 mM oleate has been used in different cell types i*n vitro* ^13, 29^, which was also used in the present study.

This study adds further evidence to distinguish the diverse function of sodium oleate and stearate. The presence of both long chain fatty acids resulted in the formation of lipid droplets in the MSC-2 cell line, however the impact on cell function was very different. Here only the presence of sodium oleate but not sodium stearate, mediated an inhibitory function in the MSC-2 cell line as well as in bone marrow-derived macrophages in the T cell proliferation assay. In line, only sodium oleate treatment led to an increase of nitric oxide (NO) production, a previously described mechanism for MDSC to suppress T cell activation and proliferation.

NO is known to be one of the most versatile players in the immune system. It has been shown to be involved in the pathogenesis and control of infectious diseases, tumors, autoimmune processes and chronic degenerative diseases^30^. NO has been considered pro-inflammatory for its anti-microbial effect and at the same time anti-inflammatory for its regulatory capacity^30^. Macrophage-derived NO suppresses T cell activation via a reversible disruption of the Jak3/STAT5 signaling pathway^31^ and MDSC-derived NO has been described to suppress T cell proliferation by blocking the IL-2 signaling pathway^32^. The IFNγ signaling pathway is well characterized as an important pathway initiatiating NO production. In this manuscript, we also found that IFNγ neutralization blocked the oleate induced suppressive phenotype of MSC-2 cells, indicating an essential role of IFNγ on the inhibitory function of myeloid cells. However, downstream events of the IFNγ signaling pathway, including phosphorylation of STAT1 and STAT3, were not altered by oleate treatment (Supp. Figure 5). Interestingly, IFNγ stimulation elevated the expression of IFNγ receptor CD119 on RAW264.7 but not MSC-2 cell line (Supp. Figure 5). According to the significant upregulation of iNOS expression (Fig. 3E) and NO production in both, cell line and primary myeloid cells, after oleate treatment, we conclude that IFNγ acts as an initiator but not an oleate associated regulator for NO production. In other words, IFNγ signaling does not regulate the concentration of NO after starting the production in myeloid cells. This conclusion can be supported by the quantification of IFNγ in the supernatants of T cell - myeloid cell cocultures, where oleate pretreated myeloid cells show a decreased amount of IFNγ accompanied with an increased amount of NO. And both, TOFA and DGAT inhibitors, which could block lipid droplet formation, also impaired the production of NO in MSC-2 cell line and primary myeloid cells. Suggesting that the NO mediated immune suppression after the initiation of IFNγ is controlled by the lipid metabolism.

Phenotypically, the induction of an inhibitory phenotype in MSC-2 cells was accompanied by the formation of lipid droplets in our model. Previous studies in tumor immunology revealed that the accumulation of lipid droplets in dendritic cells impaired their ability of antigen cross presentation, which in turn promoted tumor progression^13^. Here, the reduction of lipid droplet formation via TOFA, contributed to the recovery of antigen presentation in dendritic cells. While we could confirm a correlation between lipid droplet formation in MSC-2 cells and an increase in the suppressive capacity, the data from sodium stearate treatment are contradictory. This led to question, whether lipid droplet formation itself or rather a metabolic effect, introduced by the presence of a defined FFA mediates the regulatory phenotype. As described above, TOFA inhibits *de novo* fatty acid synthesis, hence it directly affects the FFA concentration in the cytoplasm. However, this does not allow for distinguishing in between the effect of FFA or lipid droplet formation in the cytoplasm. Inhibition of DGAT1 and DGAT2, both are required for the formation of lipid droplets^21^, allows for confining the effect of FFA *versus* lipid droplets. Thus the experiments with DGAT1&2 inhibitors clearly proved that the suppressive phenotype depends on the formation of lipid droplets in the cells.

In contrast to tumor cells which are able to synthesize and release fatty acids, immune cells prefer the uptake of exogenous fatty acids^20, 33^. According to our data, the inhibition of *de novo* fatty acid synthesis by TOFA proved less efficient than inhibition of DGAT1/2. Thus we consider the presence and incorporation of exogenous fatty acids as critical for the functional induction of myeloid suppressor cells. Blocking the induction of regulatory cells by DGAT inhibitors should be considered as a novel therapeutic strategy for solid tumors.

However, the analysis of primary CD11b^+^ cells isolated from the tumor site of tumor-bearing mice, indicate that exogenous FFA cannot further increase the inhibitory function of already immunosuppressive cells (Supp. Figure 2). This implies that myeloid cells from the tumor site are either already saturated with exogenous FFA or represent mature cells, which simply cannot be shaped into a regulatory phenotype.

There are several published protocols to generate myeloid suppressor cells *in vitro* ^22, 34^. In all the protocols, GM-CSF performed as a central cytokine during the differentiation of myeloid cells into immunosuppressive cells. In contrast to the time course for MDSC induction (3–4 days), in our hands GM-CSF was sufficient to induce the polarization of CD206^+^ macrophages in seven days (data not shown). With this protocol, an accumulation of Gr1^+^ cells could be found after oleate treatment, but this population did not exhibit a potent immunosuppressive capacity (Fig. 7; Supp. Figure 4). Therefore, although CD11b and Gr1 are widely used as MDSC markers, we would not consider them as MDSC. On the other hand, our protocol indeed induced strong immunosuppressive cells from bone marrow in the presence of oleate but not stearate. But as the sorting experiment demonstrated, the Gr1^−^ cells are actually the ones which occupy the dominant place in oleate induced inhibition of primary T cells. This Gr1^−^ population shows an about 10 fold higher suppressive capacity compared to the Gr1^+^ populations. Interestingly, the data from Gr1^−^ cells matched exactly all the findings in the MSC-2 cell line. Although MSC-2 is a well-accepted cell line to represent MDSC, the original paper from Apolloni *et al*. addressed a phenotype of MSC-2 between MDSC and tumor associated macrophages, as proved by the loss of Gr1 expression as well as an upregulation of F4/80^35^. The expression of F4/80 on Gr1^−^ cells after oleate treatment illustrated the macrophage phenotype. Furthermore, we found an elevated level of CD163 and CD206 in oleate treated Gr1^−^ cells compared with either BSA incubated Gr1^−^ cells or oleate treated Gr1^+^ cells. These data implied the characterization of Gr1^−^ cells as M2- like tumor associated macrophages (Fig. 7; Supp. Figure 4). In line, both oleate induced lipid droplets formation and DGAT inhibitor mediated inhibition of lipid droplets were confirmed in Gr1^−^ but not Gr1^+^ subsets. Previous data from Camell *et al*. demonstrated that high oleic diet increases the expression of CD206 on macrophages isolated from mesenteric adipose tissue^36^. Meanwhile, tissue resident macrophages, for instance Kupffer cell, have been reported to explain an elevated protein level of CD206 after oleate but not palmitate treatment *in vitro* ^27^. All these data illustrate the potent effect of oleate on either bone marrow precursor cells or mature macrophages. Thus, we concluded that oleate induces a change in the bone marrow derived myeloid cells into an anti-inflammatory macrophage phenotype, paralleled by a potent immunosuppressive capacity.

Cytokine profiling of the purified Gr1^−^ populations (Fig. 7E) tells us that both, IL-6 and TNFα, which are related to tumor associated macrophages, were enriched in only oleate but not in the control or stearate group, implying a strong relationship between oleate metabolism and the formation of tumor associated macrophages in cytokine level. On the other hand, for IL-1β and IL-10, which are classified as markers for anti-inflammatory macrophages and MDSCs, there is no difference between the stearate and the oleate group. This tells us, that FFA treatment pushes the cells to an anti-inflammatory phenotype, while oleate treated cells specifically are distinguishable not only functionally but also phenotypically (surface marker as well as cytokine production). The neglectable amount of IL-4 in these myeloid cells confirmed again, that the oleate induced suppression is independent of this cytokine. Besides, none of the myeloid cells in the experiment could produce IFNγ, consistent with the conclusion that T cell derived IFNγ is responsible for the initiation of NO production.

With the present study we further elucidate previous data on the tumor-promoting effect of an oleate-enriched diet and propose potential consequences of free fatty acid release by tumor cells in the tumor microenvironment. Our data not only reveal a specific effect for sodium oleate and linoleate on myeloid precursors but in addition provide a mode of action how the presence of unsaturated fatty acids affects tumor escape by the functional induction of myeloid suppressor cells.

## Material and Methods

### Mice and cell lines

All animal protocols were approved by the regional animal study committee of Berlin (Germany, LaGeSo) and all methods were performed in accordance with the relevant guidelines and regulations as published by the study committee.

Six- to eight-week-old female BALB/c mice were obtained from Harlan Winkelmann (Borchen, Germany). The MSC-2 cell line was kindly provided by Professor Ghiringhelli (Institut National de la Santé et de la Recherche Médicale (INSERM) U866, Dijon, France). Cells were passaged at 37 °C in a 5% CO_2_ atmosphere in Dulbecco’s modified Eagle’s medium supplemented with 10% fetal bovine serum (Invitrogen GmbH, Darmstadt, Germany) and penicillin/streptomycin (100 U/ml/ 100 µg/ml, Sigma-Aldrich Chemie GmbH, Munich, Germany). The E.G7 OVA cell line was bought from ATCC (ATCC ^®^CRL-2113^TM^). The RAW264.7 cell line was passaged in the same condition as MSC-2 cell line in the presence of 1 mM sodium pyruvate. T cells were cultured in RPMI medium supplemented with 10% fetal bovine serum (Invitrogen GmbH), penicillin/streptomycin (100 U/ml/ 100 µg/ml, Sigma-Aldrich Chemie GmbH) and 50 µM β-mercaptoethanol (Sigma-Aldrich Chemie GmbH).

### Fatty acid-albumin preparation

All fatty acid salts were solubilized in water. Fatty acid salts were dissolved in hot distilled water and added rapidly to proper cell culture medium with fatty acid-free BSA at a molar ratio of 8:1 (fatty acid salt: albumin). Fatty acid-albumin complex solutions were freshly prepared prior to each experiment.

### Antibodies and chemicals

If not indicated otherwise, all chemicals were obtained from Sigma-Aldrich Chemie GmbH. The following antibodies were applied for Western blot analyses: polyclonal anti-NOS (pan) (Cell Signaling Technology, Leiden, The Netherlands) and anti-β-actin (clone AC-15) and horseradish peroxidase-labeled polyclonal rabbit anti-mouse and polyclonal goat anti-rabbit antibodies (Dako, Hamburg, Germany).

The following antibodies were applied for flow cytometric analysis: PE-CD80 (clone 16-10A1) and APC-Cy7-CD11b (clone M1/70) from BD Bioscience (Heidelberg, Germany); APC-MHCII (clone M5/114.15.2), PE-Cy7-Gr1 (clone RB6-8C5), Percp-Cy5.5-CD11c (clone N418) and PE-Cy7-CD11b (clone M1/70) are from eBioscience (Frankfurt, Germany); Percp-Cy5.5-CD4 (clone GK1.5), APC-CD4 (clone GK1.5) are from Biolegend GmbH (Fell, Germany). BODIPY was obtained from Life Technologies (Carlsbad, CA, USA).

### Inhibition of lipid droplets formation

The combination of diacylglycerol acyltransferase DGAT inhibitors (iDGAT) (DGAT 1 inhibitor: A922500; DGAT2 inhibitor: PF-06424439, both Sigma-Aldrich Chemie GmbH) served to block the lipid droplets formation. MSC-2 cells were pre-incubated with 100 nM iDGAT for 1 h, and then cultured with the indicated FFA in the presence of 100 nM iDGAT for additional 24 h.

### Primary CD11b^+^ cell isolation

Mice were subcutaneously grafted with 4 × 10^6^ MCA203 cells. After either 7 or 14 days, cells from control and tumor bearing mice were sorted as described below. Briefly, tumor tissues were bathed in 70% isopropanol for 30 s and then transferred to a Petri dish. Tumors were minced into pieces <3 mm in diameter and digested in 2 mg/ml collagenase type D at 37 °C for 1 h. The digested tissue pieces were then pressed through a 100 µm Falcon^®^ nylon cell strainer (Corning, NY, USA). Spleen single cell suspension was prepared by standard procedures. Purified cell suspensions were incubated with PE-Cy7-labeled anti-CD11b antibodies for 10 min on ice and sorted by FACSJazz (BD Bioscience). CD11b^+^ cells were subsequently cultured at the indicated concentrations in the presence of either BSA (the same amount as used for sodium oleate) or 0.2 mM sodium oleate in 96-well plates for 24 h and then co-cultured with 3 × 10^5^ purified CD4^+^ T cells to analyze T cell proliferation as described below.

### Bone marrow-derived myeloid cells

Bone marrow cells were isolated as previously described^37^. Briefly, the cavities of femur and tibia bones of BALB/c mice were flushed with PBS. The single cell suspensions were cultured in high glucose DMEM (4.5 g/L D-glucose) supplemented with 10% fetal calf serum, 100 U/ml penicillin, 100 µg/ml streptomycin and 40 ng/ml granulocyte macrophage colony-stimulating factor (GM-CSF, Peprotech, Hamburg, Germany). After 24 h of incubation, non-adherent macrophage progenitor cells were isolated and cultured in the presence of 10 ng/ml IL-4 (Peprotech), 0.2 mM BSA/sodium stearate or 0.2 mM BSA/sodium oleate plus 40 ng/ml GM-CSF for 7 days in 6-well plates. Medium was renewed every other day.

### Flow cytometry

Flow cytometric analyses were performed using standard procedures. In brief, 2 × 10^5^–10^8^ cells were harvested and resuspended in 50 µl PBS/0.5% BSA buffer in the presence of the indicated antibodies for 10 min on ice. For BODIPY staining, cells were labeled by 0.2 µg/ml BODIPY®493/503 (Life Technologies) and incubated in 37 °C for 15 min and washed by PBS. Data acquisition was performed using a FACSCANTO II device (BD Bioscience) and analyzed by FlowJo software (Tree Star, Inc., Oregon, USA).

### T cell suppression assay

For the T cell proliferation assay, syngeneic CD4^+^ /CD8^+^ T cells were isolated from spleens and lymph nodes of healthy mice and purified via MACS (Miltenyi Biotec, Bergisch Gladbach, Germany). The purity of CD4 and CD8 sorting was >98%. T cells were stained with 0.5 µM carboxyfluorescein succinimidyl ester (CFSE) for 10 min at 37 °C followed by thorough washing and resuspension in T cell medium. Five µg/ml Concanavalin A or 96-well plates pre-coated with 5 µg/ml anti-CD3 (clone 2C11) plus 5 µg/ml anti-CD28 (clone 37.51E1; both BD Pharmingen) served for T cell stimulation as indicated. Stimulated CFSE-labeled T cells were cultured in the presence or absence or either MSC-2 cells (pre-treated with 100 µg/ml mitomycin C in PBS for 10 min at 37 °C in a 5% CO_2_ atmosphere) or bone marrow-derived myeloid cells in various ratios as indicated. After 72 h, the cultured cells were washed and gated on lymphocytes. The CFSE dilution due to cell division was analyzed by a FACSCANTO II device (BD Bioscience) using the FlowJo software (Tree Star).

### Cytotoxicity Assay

CD8^+^ T cells from wild-type OT-1 mice were differentiated into CTLs as described before^38^. Briefly, CD8^+^ T cells were isolated from spleens of wild-type OT-1 mice and stimulated by 2 µg/ml anti-CD3, 4 µg/ml anti-CD28 and 100 U/ml rIL-2 for 48 hours. CTLs were then polarized in the presence of 100 U/ml rIL-2 and medium was refreshed every two days. Meanwhile, bone marrow derived myeloid cells were polarized as described before. After 6 to 9 days, CTLs were harvested and cocultured with purified Gr1^−^ myeloid cells from different group for 18 hours. 1 × 10^5^ CTLs were then cocultured with carboxyfluorescein succinimidyl ester (CFSE) labelled EG7-Ova T-cell lymphoma cells for 90 min. Cells were stained with Annexin V–eFluor 450 to measure apoptosis in CFSE^+^ EG7-Ova tumor cells. The relative cytotoxicity is calculated by the formula below:

$${\rm{Relative}}\,{\rm{Cytotoxicity}}=\frac{ \% \mathrm{Annexin}\,{\rm{V}}({\rm{x}})- \% \mathrm{Annexin}\,{\rm{V}}({\rm{E}}:{\rm{T}}=0:1)}{ \% \mathrm{Annexin}\,{\rm{V}}({\rm{Control}}\,{\rm{group}}\,{\rm{E}}:{\rm{T}}=2:1)- \% \mathrm{Annexin}\,{\rm{V}}({\rm{E}}:{\rm{T}}=0:1)}$$

### Nitric oxide measurements

MSC-2 cells or bone marrow-derived myeloid cells were stimulated with murine IFNγ (Peprotech) for either 24 or 48 h. NO production was measured in the supernatant as nitrite concentration using the Griess assay as described in the manufacturer’s instructions (Promega GmbH, Mannheim, Germany).

### Arginase activity detection

Arginase activity was determined by measuring the amount of urea generated from the hydrolysis of L-arginine, as described previously^39^. Briefly, isolated cells were lysed in the presence of 100 μl lysis solution (0.1% Triton X-100, 10 mM MnCl_2_, 25 mM Tris-HCl). In brief, lysate of CD11b^+^ Gr1^+^ cells was incubated with L-arginine. The reaction was stopped with 1 M sulfuric acid. Subsequently, α-isonitrosopropiophenone was added followed by boiling for 30 min. The concentration of urea was determined at 540 nm absorbance by the Infinite® F50/Robotic ELISA plate reader (TECAN, Männedorf, Switzerland). One U enzyme activity was defined as the amount of enzyme that catalyzes the formation of 1 μmol of urea per min.

### Western blot analysis

For iNOS, STAT1, pSTAT1, STAT3, pSTAT3 expression detection, 10^6^ cells were lysed in RIPA buffer (Life Technologies). Western blot was performed via standard procedure^40^. Densitometric analysis was performed with the Fuji MultiGauge software (Fujifilm, Düsseldorf, Germany).

### Oil-red-O Staining

Oil-red-O staining was performed as described previously^41^. Briefly, dye solution was prepared as follows: oil-red-O (0.5% w/v) were dissolved overnight in isopropanol. The solution was filtered and bi-distilled water was added. After 16 h at 4 °C, the precipitate was removed by filtration and the supernatant was stored at room temperature. MSC-2 cell layers were washed with PBS and fixed with 4% phosphate-buffered formaldehyde for 15 min at room temperature, stained with the oil-red-O dye solution for 1 h and subsequently washed with 70% ethanol. Cells were visualized by phase contrast microscopy (Zeiss Microimaging, Oberkochen, Germany).

### Cytokine Measurement

Supernatants from either myeloid cell-T cell co-culture system or primary bone marrow cells were used for cytokine measurement. Primary myeloid cells were incubated in the presence of GM-CSF plus FFAs for 7 days. 1 × 106 Gr1^−^ subsets were purified via MACS (Miltenyi Biotec, Bergisch Gladbach, Germany) and stimulated with 1 µg/ml LPS for 24 hours. The supernatant was collected for analysis. CBA inflammation kit (BD Pharmingen) was used in accordance with the manufacturer’s instructions. Samples were measured using a FACSCANTO II device and data were analyzed via FCAP array Software (BD Biosciences).

### Statistical Analysis

Statistical significance of differences between the experimental groups were determined by Student’s t-test or factorial analysis of variance and the respective post hoc tests (Tukey’s and Dunnett’s multiple comparisons test) using GraphPad Prism software (GraphPad Software, La Jolla, CA).

## Electronic supplementary material

Supplementary Data
