# Supplementary material for: Oleate but not stearate induces the regulatory phenotype of myeloid suppressor cells
Source: Sci Rep. 2017 Aug 8;7:7498. doi: 10.1038/s41598-017-07685-9 (PMC5548895; doi:10.1038/s41598-017-07685-9)
Supplement: Supplementary file 1 — Supplementary Data [file 41598_2017_7685_MOESM1_ESM.pdf]

Oleate but not stearate induces the regulatory phenotype of myeloid suppressor cells

Running title: Oleate effect on myeloid suppressor cells

Hao Wu[1,2,3], Carl Weidinger[1], Franziska Schmidt[1,3], Jacqueline Keye[1,3], Marie Friedrich[1,3], Cansu Yerinde[1], Gerald Willimsky[5,6], Zhihai Qin[2,4], Britta Siegmund*[1], Rainer Glauben*[1]

1. Charité - Universitätsmedizin Berlin, Medical Department for Gastroenterology, Infectious Diseases and Rheumatology, 12200 Berlin, Germany

2. The First Affiliated Hospital of Zhengzhou University, No.1 Jianshe East Road, Zhengzhou 450052, Henan Province, China

3. Free University Berlin, Department of Biology, Chemistry, and Pharmacy, 14195, Berlin, Germany

4. Key Laboratory of Protein and Peptide Pharmaceuticals, Institute of Biophysics, Chinese Academy of Sciences, Beijing 100101, China

5. Charité - Universitätsmedizin Berlin, Institute of Immunology, 13125 Berlin, Germany

6. German Cancer Research Center, 69120 Heidelberg, Germany

* These authors contributed equally to this work

Corresponding author:
Rainer Glauben, PhD

Charité - Universitätsmedizin Berlin

Medical Department for Gastroenterology, Infectious Diseases and Rheumatology

Hindenburgdamm 30

12200 Berlin, Germany

Phone: +49 30 450 514 343

Email: rainer.glauben@charite.de

Keywords: tumor associated macrophage, fatty acid, oleate, lipid droplets, T cell suppression, metabolism, MDSC

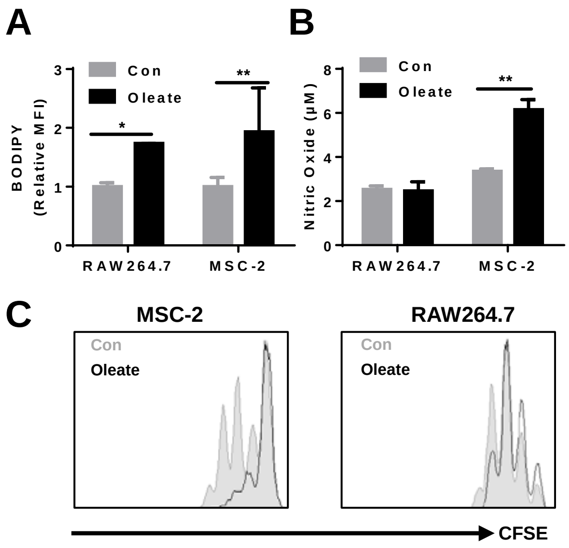

Figure S1. Sodium oleate-induced inhibitory properties are restricted to MSC-2 cells. MSC-2 or RAW264.7 cells were cultured for 24 h in the presence of BSA or sodium oleate and lipid droplets formation was determined by BODIPY staining via flow cytometry (**A**). Both cell lines were incubated as before and were then subsequently stimulated with IFNγ (10 μg/ml) for 8 h and NO production was measured by Griess reaction (**B**). Either RAW264.7 or MSC-2 cells were co-cultured with $CD4^+$ T cells in the ratios as indicated in the presence or absence of sodium oleate. Cell proliferation was evaluated by CFSE staining after 72 h (**C**). Shown are representative staining as well as mean ± SD from two to four independent experiments. * p<0.05; ** p<0.01.

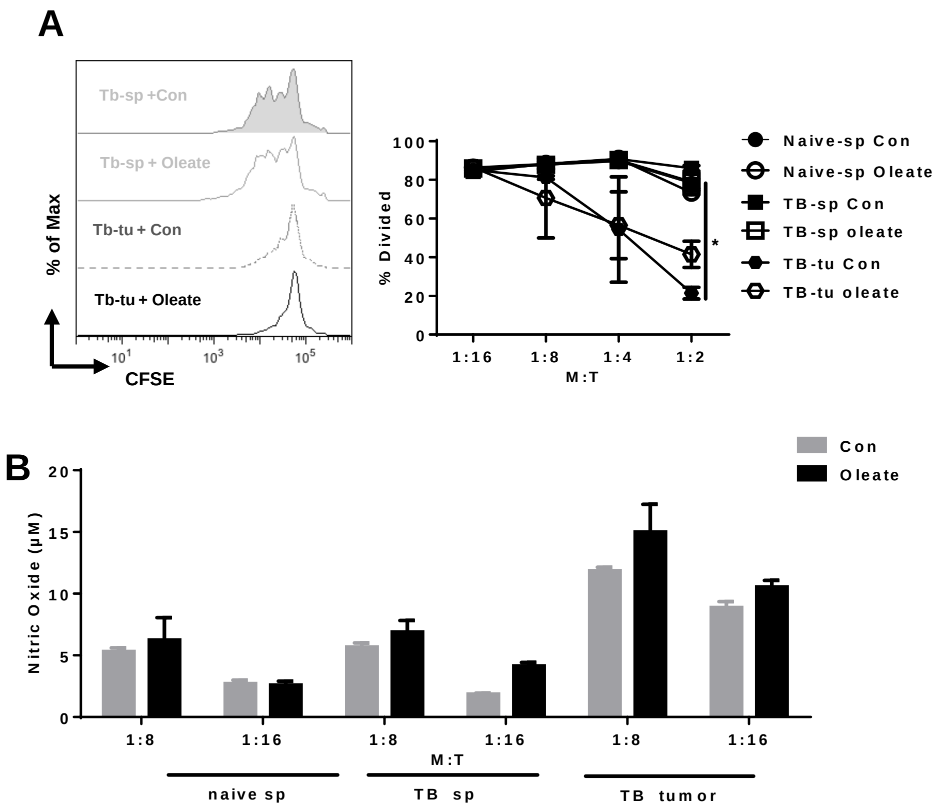

Figure S2. Sodium oleate treatment of CD11b+ cells isolated from tumor bearing mice. CD11b+ cells were isolated from spleen and tumor tissue from tumor bearing and healthy wildtype mice respectively. Cells were treated with BSA or sodium oleate (0.2 mM) for 24 h and co-cultured with CD4+ T cells in the ratios as indicated. Cell proliferation was evaluated by CFSE staining after 72 h (**A**). Supernatants of the co-culture system served for NO quantification via Griess reaction (**B**). Shown is the mean±SD from two to four independent experiments. * $p<0.05$.

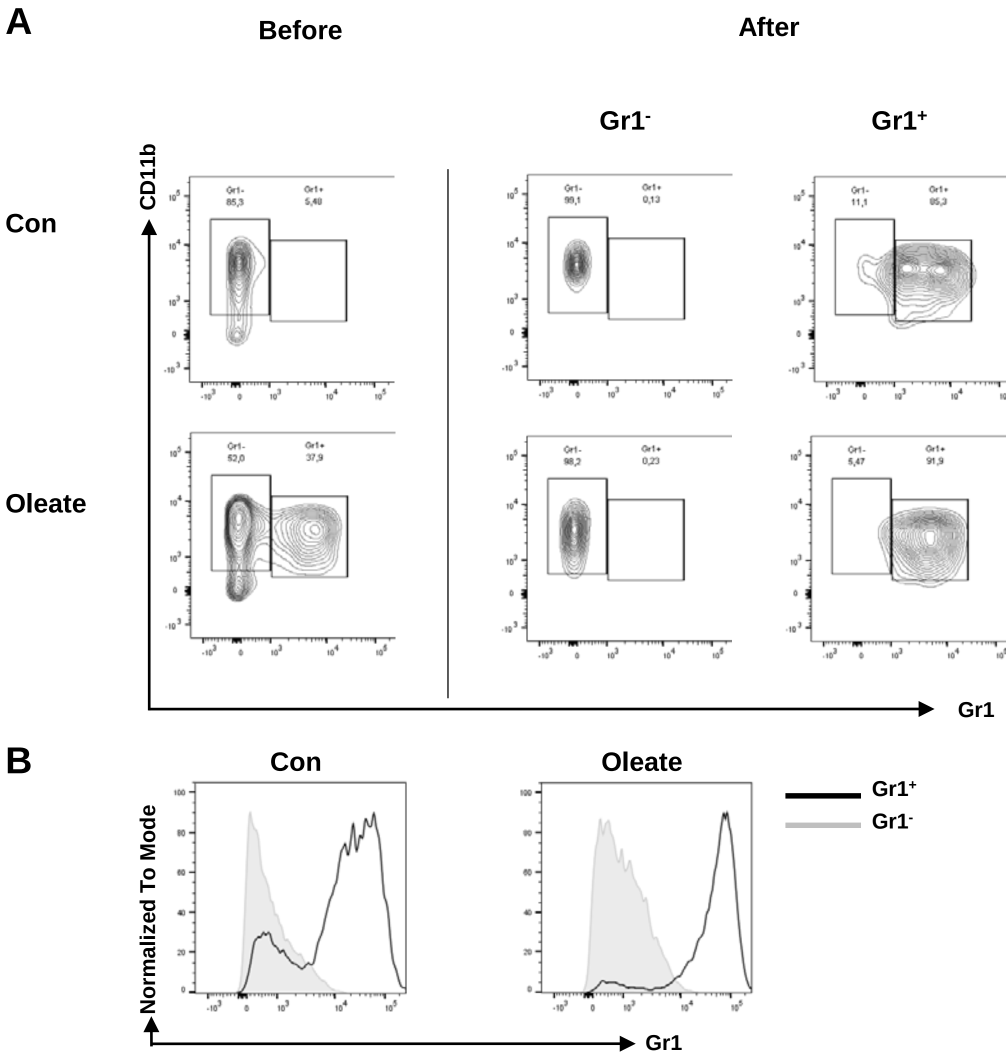

Figure S3. Gr1 sorting via flow cytometry and magnetic beads. Bone marrow cells were polarized in the presence of 40 ng/ml GM-CSF and treated with the indicated compounds for 7 days. Gr1$^+$ and Gr1$^-$ populations were purified via Flow cytometry (**A**) and magnetic beads (**B**).

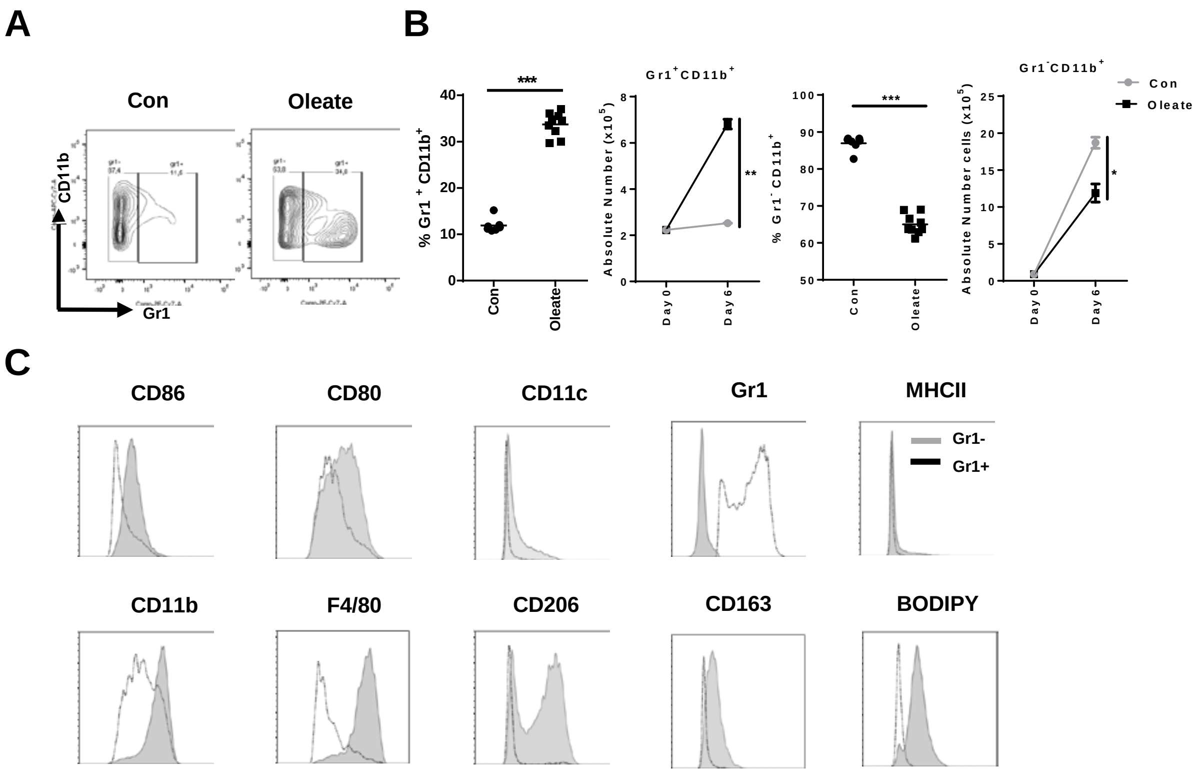

Figure S4. Sodium oleate induced accumulation of $Gr1^+$ myeloid cells in vitro. Bone marrow cells were polarized in the presence of 40 ng/ml GM-CSF and treated with the indicated compounds for 7 days. Flow cytometry was performed to detect the percentage and absolute number of $Gr1^+$ cells (A, B). $Gr1^-$ and $Gr1^+$ subsets gated from $CD11b^+$ populations was stained by indicated surface marker and acquired via FACs canto (C). Shown is the mean ± SD from two to four independent experiments. **, $p \leq 0.01$;***, $p \leq 0.001$.

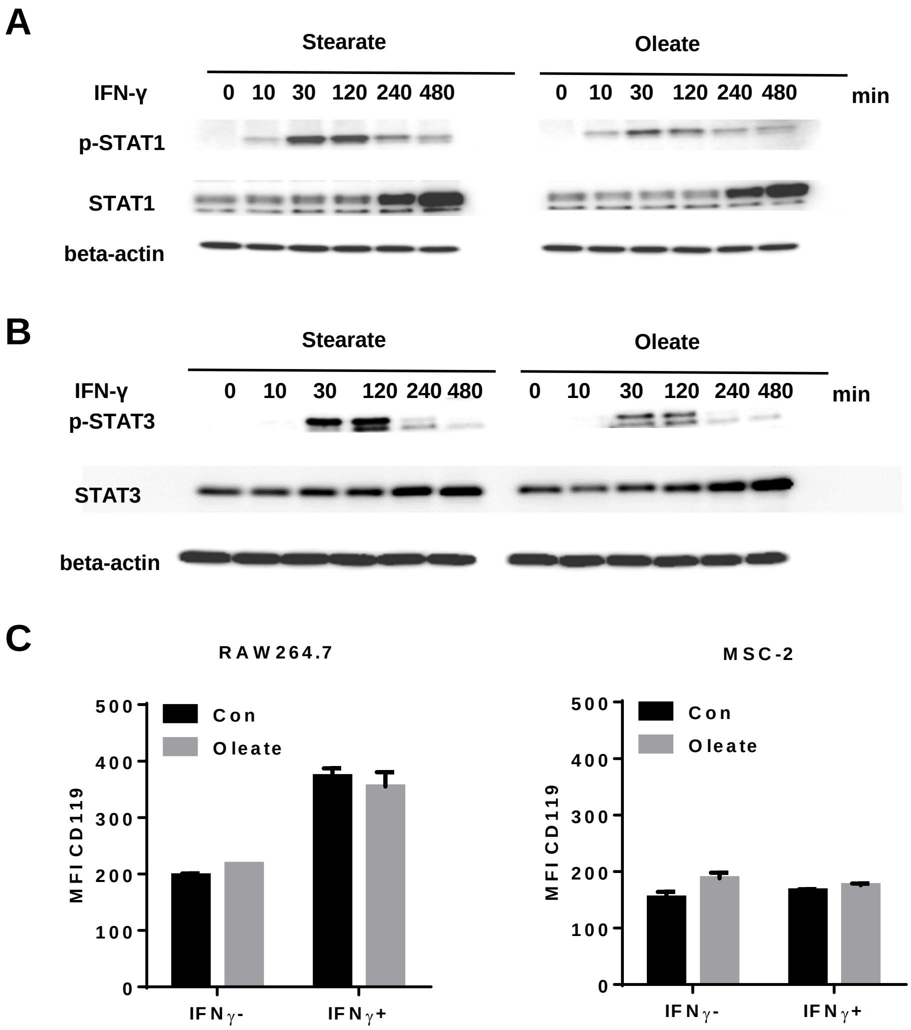

Figure S5. The effect of oleate is independent of CD119 expression and the phosphorylation of STAT1 or STAT3. MSC-2 cells was treated with FFAs for 24 hours, followed by an IFNγ stimulation (1 ng/ml) for indicated time. Cells were lysed and western blot was performed for STAT1, pSTAT1, STAT3 and pSTAT3 (A, B). Both RAW 264.7 and MSC-2 cell line were stimulated overnight in the presence of 10ng/ml IFNγ. The expression of CD119 was detected via flow cytometry (C).
